# The longitudinal relationship between foreign language learning anxiety and willingness to communicate in vocational English classrooms among Chinese students: the mediating roles of instrumental motivation and learning engagement

**DOI:** 10.3389/fpsyg.2026.1896094

**Published:** 2026-08-06

**Authors:** Qianqian Wang, Guochun Shi, Yanting Huang

**Affiliations:** 1 College of Education and Science, Bohai University, Jinzhou, China; 2 Hainan College of Foreign Studies, Wenchang, China; 3 School of Asian and European Languages, Hainan College of Foreign Studies, Wenchang, Hainan, China; 4 Department of Counseling Psychology, General Graduate School, Dongshin University, Najusi, Republic of Korea

**Keywords:** foreign language learning anxiety, instrumental motivation, learning engagement, longitudinal study, vocational English, vocational students, willingness to communicate

## Abstract

The willingness to communicate (WTC) in vocational English classrooms involves the combined roles of achievement emotions, future-oriented motivational beliefs, and classroom engagement. Drawing on control-value theory (CVT) and the L2 motivational self system (L2MSS), this study developed a three-wave longitudinal mediation model to examine the relationships among foreign language learning anxiety, instrumental motivation, learning engagement, and willingness to communicate (WTC) among Chinese non-English major vocational students. Participants (*N* = 232) from a vocational college completed three waves of surveys at the beginning, middle, and end of the semester. Foreign language learning anxiety was assessed using the 8-item S-FLCAS; instrumental motivation for vocational English was measured using an adapted version of the Instrumental Motivation subscale; learning engagement was tracked via the 9-item Language Classroom Engagement Scale; and WTC was evaluated using an adapted WTC in English scale. The longitudinal path analysis indicated that Time 1 foreign language learning anxiety was negatively associated with Time 3 WTC. Crucially, Time 2 instrumental motivation and Time 3 learning engagement each served as a significant mediator in this relationship, and sequentially formed a significant longitudinal chain mediation path. These findings indicate that initial anxiety is longitudinally related to subsequent differences in career-related instrumental motivation and classroom engagement, which are further associated with students’ willingness to communicate in vocational English classrooms. The findings highlight the importance of considering emotional experiences, vocational relevance, and classroom engagement when designing vocational English instruction.

## 1 Introduction

In the context of international industry-education integration in Chinese vocational education, researchers and language teachers often observe that many vocational students recognize the practical importance of English but remain reluctant to communicate in English classroom activities (Xu et al., 2022). On the one hand, with the upgrading of industries such as cross-border e-commerce, smart manufacturing, international tourism, and cross-cultural nursing, English is increasingly regarded as a career-related communicative skill rather than only a general academic subject (Roshid and Kankaanranta, 2025). On the other hand, students frequently show hesitation during classroom interactions, role-plays, group discussions, and simulated workplace communication tasks (Shao et al., 2019). This gap between recognizing the value of English and being willing to speak English in class suggests that vocational students’ willingness to communicate (WTC) should be examined not only as a language proficiency issue, but also as a psychological and motivational process.

Previous explanations of limited classroom communication have often emphasized what may be called a single cognitive deficit perspective. In this perspective, students’ willingness to communicate is mainly attributed to insufficient vocabulary, weak grammar knowledge, poor pronunciation, or limited general language proficiency (Mak, 2011). Although language competence is undoubtedly important, this perspective is incomplete because it gives limited attention to learners’ emotional experiences, future-oriented motivational beliefs, and active classroom engagement. Research on the psychology of language learning indicates that learners’ willingness to use a second or foreign language is shaped by multiple interacting factors, including anxiety, perceived value, motivation, engagement, classroom context, and communication confidence (Lin and Wang, 2025; MacIntyre et al., 1998; Peng and Woodrow, 2010). Therefore, understanding WTC in vocational English classrooms requires an integrated model that connects affective, motivational, and engagement-related variables over time.

Willingness to communicate in a second language was originally conceptualized by MacIntyre et al. (1998) as a readiness to enter into discourse at a particular time with a particular person or persons. Their heuristic pyramid model views L2 WTC as a layered construct that includes personality traits, intergroup climate, social and individual context, affective-cognitive context, motivational propensities, situated antecedents, and immediate communication intention. In the Chinese EFL classroom context, Peng and Woodrow (2010) further showed that classroom environment, learner beliefs, motivation, and communication confidence are associated with students’ WTC. These studies provide an important foundation for examining WTC as a situated and psychologically embedded classroom construct. However, vocational English classrooms differ from general university English classrooms because their instructional goals are closely connected with future occupational tasks, workplace communication, and professional identity development.

Compared with general higher education, English teaching in vocational colleges has a distinct application-oriented orientation and is often connected with English for Specific Purposes or English for Occupational Purposes (Gai, 2017). Vocational students are expected to use English in practical scenarios such as receiving international guests, reading workplace instructions, conducting service communication, explaining technical information, or participating in job interviews. In this context, WTC is not only related to whether students feel emotionally safe when speaking English, but also to whether they perceive vocational English as useful for their future careers and whether they are sufficiently engaged in classroom tasks. Many vocational students may acknowledge that English is valuable for employment, certification, or career advancement, yet their previous experiences of frustration in foreign language learning may make classroom communication emotionally threatening (Han and Hamzah, 2024). This suggests that foreign language learning anxiety, instrumental motivation, and learning engagement may jointly shape vocational students’ WTC.

Foreign language classroom anxiety is one of the most widely studied negative emotions in language learning. It refers to the tension, worry, nervousness, and fear associated with foreign language learning and use, especially in evaluative or communicative classroom situations (Horwitz et al., 1986; MacIntyre, 2017). Meta-analytic evidence indicates that foreign language anxiety is negatively related to language achievement (Teimouri et al., 2019). Recent longitudinal and structural studies have also shown that anxiety may be associated with changes in learners’ motivational beliefs, enjoyment, engagement, and communication-related outcomes over time (Dewaele et al., 2023; Pan and Zhang, 2023; Wang et al., 2025). For vocational students, anxiety may be especially salient because classroom speaking tasks often require immediate oral performance in front of peers and teachers. These tasks may activate concerns about making mistakes, losing face, or being judged as incompetent.

To explain the emotional foundation of the present model, this study draws on control-value theory (CVT) (Pekrun, 2006, 2024). CVT proposes that achievement emotions are closely related to learners’ appraisals of control and value in achievement settings. When learners perceive low control over a task or view the task as threatening, they are more likely to experience negative achievement emotions such as anxiety. These emotions are associated with attention allocation, motivation, engagement, and academic behavior (Pekrun, 2024). In vocational English classrooms, foreign language learning anxiety can be understood as an achievement-related emotional experience that reflects students’ perceived difficulty in controlling classroom communication demands. Students who feel anxious at the beginning of the semester may enter later classroom activities with reduced emotional security and lower confidence in handling English communication tasks.

While CVT helps explain the emotional component of the model, the L2 motivational self system (L2MSS) provides a framework for understanding the future-oriented motivational component (Dörnyei, 2009). L2MSS emphasizes that language learning motivation is linked to learners’ future self-guides, including who they hope to become as language users. In vocational education, students’ future L2 self is often connected with professional roles, employability, workplace communication, and career development. Therefore, instrumental motivation is particularly relevant to vocational English learning because it captures students’ perceived practical benefits of learning English for future occupational goals. In the present study, instrumental motivation refers to the extent to which students perceive vocational English as useful, important, and relevant to their future career and workplace needs (Taguchi et al., 2009).

The relationship between anxiety and instrumental motivation is theoretically important. From the perspective of CVT, students’ emotional experiences are associated with how they evaluate academic tasks and outcomes. From the perspective of L2MSS, students’ motivation is linked to whether language learning is meaningfully connected with future identities and goals. When students experience strong anxiety in vocational English classrooms, they may come to view classroom communication less as a pathway toward future professional development and more as a source of psychological pressure. In this process, anxiety may be negatively associated with students’ later instrumental motivation. For example, a student who repeatedly feels nervous during English role-plays may gradually question whether vocational English is useful or attainable, even if the course is objectively relevant to employment. Thus, instrumental motivation may function as an important mediator linking early anxiety with later WTC.

Learning engagement provides a further link between motivational beliefs and classroom communication. Fredricks et al. (2004) conceptualized engagement as a multidimensional construct that includes behavioral, emotional, and cognitive engagement. In language learning, engagement reflects not only whether students participate in classroom activities, but also whether they show interest, invest effort, think deeply about learning tasks, and use strategies to complete communication activities (Eerdemutu et al., 2024; Hiver et al., 2024). For vocational English learners, engagement can be observed in activities such as listening carefully to instructions, participating in role-plays, connecting classroom content with workplace situations, and attempting to express professional ideas in English. These engagement behaviors and experiences are closely related to students’ readiness to communicate because WTC requires both psychological willingness and task-based participation.

The connection between instrumental motivation and engagement is also central to vocational English learning. Students who believe that vocational English is useful for their future careers may be more willing to invest effort in classroom tasks, especially when these tasks resemble real workplace situations. Conversely, students with lower instrumental motivation may treat classroom activities as disconnected from their future goals and may participate only passively. Recent research on language learning engagement suggests that motivation needs to be translated into active behavioral, emotional, and cognitive participation before it can be associated with language learning outcomes (Henry et al., 2024; Hiver et al., 2024; Zare et al., 2025). Therefore, learning engagement may serve as a proximal mediator between instrumental motivation and WTC.

A review of the existing literature reveals three main gaps. First, many empirical studies on WTC in foreign language classrooms rely on cross-sectional data, which limits the ability to examine how emotional, motivational, and engagement-related variables are associated with later WTC across a teaching cycle (Pan and Zhang, 2023). Second, most existing models focus on general university students and do not sufficiently consider the vocational education context, where instrumental motivation related to career development is especially salient. Third, although previous studies have examined anxiety, motivation, engagement, and WTC separately, few have tested a longitudinal chain mediation model that links these variables within one integrated framework. As a result, the process through which early foreign language learning anxiety is associated with later instrumental motivation, engagement, and WTC among vocational students remains insufficiently understood.

To address these gaps, the present study integrates CVT and L2MSS to construct a three-wave longitudinal mediation model. CVT explains why foreign language learning anxiety may be associated with later motivational and engagement-related differences, while L2MSS explains why instrumental motivation is a meaningful future-oriented motivational construct in vocational English learning. This integrated framework allows the study to examine whether anxiety at the beginning of the semester is associated with WTC at the end of the semester through instrumental motivation in the middle of the semester and learning engagement at the end of the semester. By focusing on Chinese non-English major vocational students, this study also extends WTC research to a learner group whose English learning is strongly connected with occupational preparation and practical workplace communication.

Based on this theoretical rationale, the study proposes the following hypotheses:

*H1*: Foreign language learning anxiety is significantly and negatively associated with willingness to communicate in vocational English classrooms.

*H2*: Instrumental motivation mediates the relationship between foreign language learning anxiety and willingness to communicate.

*H3*: Learning engagement mediates the relationship between foreign language learning anxiety and willingness to communicate.

*H4*: Instrumental motivation and learning engagement sequentially mediate the relationship between foreign language learning anxiety and willingness to communicate.

## 2 Method

### 2.1 Study design

This study adopted a three-wave longitudinal survey design to examine the relationships among foreign language learning anxiety, instrumental motivation, learning engagement, and willingness to communicate in vocational English classrooms. Data were collected during the second semester of the 2025–2026 academic year. The three waves corresponded to the beginning, middle, and end of the semester.

Time 1 was conducted in Week 2 and was used to measure students’ foreign language learning anxiety and baseline variables. Time 2 was conducted in Week 8 and focused on intermediate motivational variables, especially instrumental motivation for vocational English. Time 3 was conducted in Week 15 and measured learning engagement and willingness to communicate in vocational English classrooms. The interval between adjacent waves was approximately 6 weeks. This interval was selected to capture changes across the teaching cycle while reducing possible test–retest effects associated with very short intervals (Selig and Preacher, 2009).

A time-separated design was used to reduce the potential influence of common method bias and to strengthen the temporal ordering of the proposed mediation model. To improve the robustness of the analysis, prior levels of key variables were controlled in the structural model. Specifically, the model included autoregressive controls from Time 1 instrumental motivation to Time 2 instrumental motivation, from Time 2 learning engagement to Time 3 learning engagement, and from Time 1 willingness to communicate to Time 3 willingness to communicate.

### 2.2 Participants

Participants were non-English major students from a vocational college in southern China. We initially distributed 300 questionnaires. After matching the responses across the three waves, we obtained 232 valid questionnaires. Attrition analysis showed that attrited and retained participants did not differ significantly on any of the Time 1 variables (all t < 1.52, *p* > 0.13), ruling out systematic attrition bias. The valid matching rate was 77.3%. The final sample included 153 female students (Code = 0) and 79 male students (Code = 1). Gender was included as a control variable in the path model. Students also reported their self-reported English proficiency by selecting one of two options: weak (Code = 0) or good (Code = 1). In the final sample, 155 students reported weak self-reported English proficiency, while 77 reported good self-reported English proficiency. Self-reported English proficiency was included as a control variable because prior language proficiency may be related to anxiety, engagement, and willingness to communicate.

### 2.3 Ethical procedures

The Institutional Review Board of Hainan Medical University approved this study (Ethics Approval Number: HYMLL-2025-035). Before each data collection session, the researchers explained the study’s purpose, the principle of voluntary participation, and data confidentiality measures to the participants. Participants could only join the study after signing an informed consent form. The questionnaires were distributed online through the Wenjuanxing platform. Each survey took about 10 to 15 min to complete.

### 2.4 Measures

All constructs were measured using established or adapted scales. The questionnaire consisted of five sections: basic information, foreign language classroom anxiety, instrumental motivation, learning engagement, and willingness to communicate. All scales followed a standard translation and back-translation procedure. Their semantic equivalence and contextual appropriateness were checked through expert review and a small-scale pilot test before formal data collection.

Foreign Language Classroom Anxiety. This construct was measured using the 8-item Short-form Foreign Language Classroom Anxiety Scale (S-FLCAS) validated by Botes et al. (2022a,b). A sample item is “I feel nervous when I am preparing to answer a question in English class.” Responses were recorded on a 5-point Likert scale ranging from 1 (strongly disagree) to 5 (strongly agree). The Cronbach’s alphas for this scale across the three waves in this study were 0.891, 0.888, and 0.901.

Instrumental Motivation in Vocational English. This construct was measured using an adapted version of the Instrumental Motivation subscale from the L2 Motivational Self System (Dörnyei, 2009; Taguchi et al., 2009). This subscale assesses the extent to which students perceive language learning as a practical and useful asset for their future career advancement and professional goals. The Chinese version of the items was derived from the validated Chinese questionnaire used by Taguchi et al. (2009), with the word “English” replaced by “vocational English” to ensure contextual appropriateness for vocational college students. For example, an item reads, “Learning vocational English is necessary for my future career advancement and promotion.” The subscale includes 5 items rated on a 6-point Likert scale. Higher scores indicate that students experience higher instrumental motivation regarding vocational English for their future careers. The Cronbach’s alphas across the three waves were 0.857, 0.879, and 0.850.

Learning Engagement. We used the 9-item short-form Language Classroom Engagement Scale developed by Eerdemutu et al. (2024) for foreign language classroom contexts. This scale covers behavioral, emotional, and cognitive engagement. It uses a 5-point Likert scale. For example, one item stated, “I actively participate in group discussions, role-plays, or vocational tasks in class.” The research sample in Eerdemutu et al. (2024) included Chinese language learners, and the findings supported the scale’s construct validity. Higher scores reflect a higher level of student engagement in the vocational English classroom. The Cronbach’s alphas for the three waves were 0.913, 0.901, and 0.925.

Willingness to communicate in vocational English was measured using an adapted 10-item scale based on the WTC in English scale by Peng and Woodrow (2010). The scale assessed students’ inner willingness to communicate in English in specific vocational English classroom situations. The items were adapted to fit vocational classroom tasks such as workplace role-plays, group discussions, project presentations, job interview simulations, and classroom problem-solving activities.

Sample items included “Volunteer to answer the teacher’s questions in English,” “Participate in a role-play simulating a specific workplace scenario in English,” and “Present your group’s vocational task results or project to the whole class in English.” The questionnaire instructions asked students to rate their willingness to communicate even if they felt that their spoken English was not perfect.

It uses a 6-point Likert scale. Higher scores indicated stronger willingness to communicate in vocational English classroom situations. The Cronbach’s alpha values across the three waves were 0.921, 0.925, and 0.911, respectively.

### 2.5 Data analysis

The data analysis involved six steps:

First, common method bias was examined using Harman’s single-factor test. An unrotated principal component analysis was conducted for each wave of data. If the first factor explained less than 40% of the total variance, common method bias was considered not to be a serious concern (Podsakoff et al., 2003).

Second, reliability and validity were assessed. Cronbach’s alpha and McDonald’s omega were calculated to evaluate internal consistency. Confirmatory factor analysis (CFA) was conducted to test construct validity. Composite reliability (CR) and average variance extracted (AVE) were calculated to evaluate convergent validity. Discriminant validity was examined by comparing the square root of AVE with the correlations among constructs.

Third, descriptive statistics and Pearson correlation coefficients were calculated for the core variables. Means, standard deviations, minimum values, and maximum values were reported.

Fourth, longitudinal path analysis was conducted using observed variable path modeling. Mean scores were used as observed indicators. The maximum likelihood robust (MLR) estimator was applied to estimate the model. The main model included Time 1 foreign language learning anxiety as the predictor, Time 2 instrumental motivation and Time 3 learning engagement as mediators, and Time 3 willingness to communicate as the outcome. Gender and self-reported English proficiency were included as control variables. Autoregressive paths were included to control for prior levels of key constructs.

Fifth, mediation effects were tested using the bootstrap method with 5,000 resamples. Bias-corrected percentile confidence intervals were calculated. A 95% confidence interval that did not include zero was interpreted as evidence of a significant mediation effect (Hayes, 2018).

Sixth, robustness tests were conducted by comparing the main model with alternative models and reverse path models. Alternative models included single-mediator models that retained only instrumental motivation or only learning engagement. Reverse path tests were used to examine whether earlier willingness to communicate or instrumental motivation was associated with later foreign language learning anxiety after controlling for prior anxiety. Variance inflation factors were also examined to assess multicollinearity.

All analyses were conducted using R version 4.3.0. The lavaan package was used for structural equation modeling, and the psych package was used for reliability and factor analyses.

## 3 Results

### 3.1 Common method bias and measurement model

As shown in Table 1, Harman’s single-factor tests for each wave indicated that the first factor explained 20.00, 23.48, and 28.88% of the variance for Time 1, Time 2, and Time 3, respectively. All these values are below the 40% empirical threshold.

**Table 1:** Common method bias and measurement model fit.

Time	Harman’s 1st factor Variance	χ^2^(df)	*p*	CFI	TLI	RMSEA	SRMR	Single-factor CFI	ΔCFI
T1	20.00%	488.20 (458)	0.159	0.992	0.991	0.017	0.048	0.239	0.752
T2	23.48%	506.32 (458)	0.059	0.987	0.985	0.021	0.045	0.403	0.584
T3	28.88%	566.32 (458)	<0.001	0.972	0.969	0.032	0.047	0.470	0.501

Further CFA results showed that the four-factor models fit the data well across all three waves. They were notably better than the single-factor models. The fit indices for the Time 1 four-factor model were *χ*^2^(458) = 488.20, *p* = 0.159, CFI = 0.992, TLI = 0.991, RMSEA = 0.017, and SRMR = 0.048. For the Time 2 four-factor model, the indices were *χ*^2^(458) = 506.32, *p* = 0.059, CFI = 0.987, TLI = 0.985, RMSEA = 0.021, and SRMR = 0.045. For the Time 3 four-factor model, the indices were *χ*^2^(458) = 566.32, *p* < 0.001, CFI = 0.972, TLI = 0.969, RMSEA = 0.032, and SRMR = 0.047. The CFI values for the single-factor models across the three waves were noticeably lower. This indicates good discriminant validity among the four constructs.

### 3.2 Reliability and convergent validity

Table 2 shows that all scales demonstrated good internal consistency across the three waves of data. The Cronbach’s alpha values ranged from 0.850 to 0.925. The McDonald’s omega values also ranged from 0.850 to 0.925. Furthermore, the composite reliability (CR) scores were all above 0.850, and the average variance extracted (AVE) scores were all above 0.500. These results indicate that the measurement tools possess satisfactory reliability and convergent validity for all four constructs across the three time points.

**Table 2:** Reliability, composite reliability, and AVE.

Time	Construct	*α*	*ω*	CR	AVE
T1	Foreign language anxiety	0.891	0.891	0.892	0.509
T1	Instrumental motivation	0.857	0.857	0.858	0.547
T1	Learning engagement	0.913	0.913	0.913	0.539
T1	Willingness to communicate	0.921	0.921	0.921	0.540
T2	Foreign language anxiety	0.888	0.888	0.888	0.501
T2	Instrumental motivation	0.879	0.879	0.880	0.595
T2	Learning engagement	0.901	0.901	0.902	0.506
T2	Willingness to communicate	0.925	0.925	0.925	0.552
T3	Foreign language anxiety	0.901	0.901	0.901	0.533
T3	Instrumental motivation	0.850	0.850	0.850	0.533
T3	Learning engagement	0.925	0.925	0.925	0.580
T3	Willingness to communicate	0.911	0.911	0.912	0.510

### 3.3 Descriptive statistics and correlation analysis

Table 3 presents the descriptive statistics for all variables. The mean score for Time 1 foreign language learning anxiety was 3.203 out of 5. The mean for Time 2 instrumental motivation for vocational English was 3.688 out of 6. The mean for Time 3 learning engagement was 3.071 out of 5, and the mean for Time 3 willingness to communicate in the vocational English classroom was 3.545 out of 6. Overall, vocational students reported a moderate to high level of instrumental motivation for vocational English. However, their learning engagement and classroom willingness to communicate did not reach a high level. This suggests that while students recognize the practical value of vocational English to some extent, this recognition does not always correspond with active classroom participation and communication.

**Table 3:** Descriptive statistics of variables.

Variable	*N*	*M*	SD	Min	Max
T1 foreign language anxiety	232	3.203	0.772	1.125	4.750
T1 instrumental motivation	232	3.872	0.951	1.600	5.800
T1 learning engagement	232	3.092	0.772	1.222	4.778
T1 willingness to communicate	232	3.338	0.931	1.300	5.600
T2 foreign language anxiety	232	3.236	0.767	1.125	5.000
T2 instrumental motivation	232	3.688	1.002	1.200	6.000
T2 learning engagement	232	3.139	0.751	1.222	4.778
T2 willingness to communicate	232	3.478	0.957	1.400	5.700
T3 foreign language anxiety	232	3.236	0.768	1.500	5.000
T3 instrumental motivation	232	3.784	0.962	1.600	6.000
T3 learning engagement	232	3.071	0.790	1.000	4.778
T3 willingness to communicate	232	3.545	0.902	1.700	5.400

Table 4 displays the correlation matrix for the core variables. The results revealed significant negative relationships between Time 1 foreign language learning anxiety and subsequent variables. It was negatively correlated with Time 2 instrumental motivation (r = −0.313, *p* < 0.001), Time 3 learning engagement (*r* = −0.328, *p* < 0.001), and Time 3 willingness to communicate (r = −0.406, *p* < 0.001). Conversely, Time 2 instrumental motivation showed significant positive correlations with Time 3 learning engagement (*r* = 0.390, *p* < 0.001) and Time 3 willingness to communicate (*r* = 0.387, *p* < 0.001). Additionally, Time 3 learning engagement was positively associated with Time 3 willingness to communicate (*r* = 0.429, *p* < 0.001). These correlations align with the proposed theoretical directions.

**Table 4:** Correlation matrix of core variables.

Variable	FLA1	IM1	IM2	LE2	LE3	WTC1	WTC3
FLA1	1.000						
IM1	−0.193**	1.000					
IM2	−0.313***	0.425***	1.000				
LE2	−0.066	0.127	0.067	1.000			
LE3	−0.328***	0.285***	0.390***	0.463***	1.000		
WTC1	−0.084	0.078	0.169**	0.227***	0.098	1.000	
WTC3	−0.406***	0.292***	0.387***	0.171**	0.429***	0.319***	1.000

FLA, Foreign Language Anxiety; IM, Instrumental Motivation; LE, Learning Engagement; WTC, Willingness to Communicate. *p < 0.05, **p < 0.01, ***p < 0.001.

### 3.4 Longitudinal path analysis

**Figure 1:**
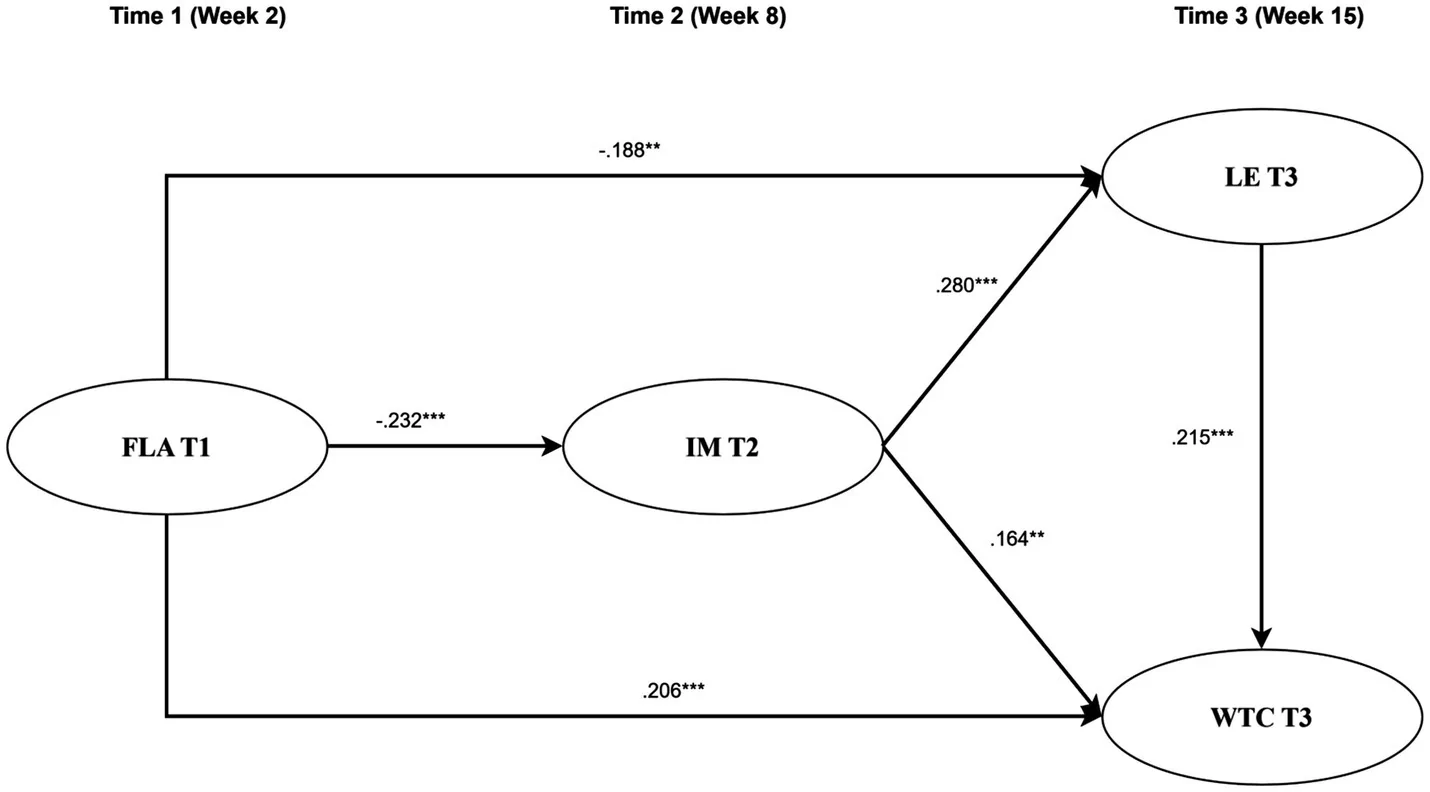
The longitudinal model of anxiety, motivation, engagement, and WTC.

The main model demonstrated a good fit to the data: *χ*^2^(6) = 6.41, *p* = 0.378, CFI = 0.999, TLI = 0.993, RMSEA = 0.017, and SRMR = 0.017. As shown in Table 5 and Figure 1, the structural path analysis indicated that Time 1 foreign language learning anxiety was negatively associated with Time 2 instrumental motivation for vocational English (*B* = −0.301, *β* = −0.232, *p* < 0.001). Time 2 instrumental motivation was positively associated with Time 3 learning engagement (*B* = 0.221, *β* = 0.280, *p* < 0.001). Time 3 learning engagement was positively associated with Time 3 willingness to communicate in the classroom (*B* = 0.246, *β* = 0.215, *p* < 0.001). Time 2 instrumental motivation also showed a positive relationship with Time 3 willingness to communicate (*B* = 0.148, β = 0.164, *p* = 0.002). After controlling for autoregressive paths, gender, and English proficiency, the direct relationship between Time 1 foreign language learning anxiety and Time 3 willingness to communicate remained significant (*B* = −0.241, *β* = −0.206, *p* < 0.001). Furthermore, the R^2^ results indicated that the model explained 24.1% of the variance in Time 2 instrumental motivation, 40.4% of the variance in Time 3 learning engagement, and 42.4% of the variance in Time 3 willingness to communicate. This suggests that the model has adequate explanatory power.

**Table 5:** Results of longitudinal path analysis.

Path	*B*	*β*	SE	*p*
T1 FLA → T2 IM	−0.301	−0.232	0.075	0.0001
T1 IM → T2 IM	0.393	0.373	0.062	<0.001
T2 IM → T3 LE	0.221	0.280	0.039	<0.001
T1 FLA → T3 LE	−0.192	−0.188	0.058	0.0009
T2 LE → T3 LE	0.455	0.433	0.054	<0.001
T2 IM → T3 WTC	0.148	0.164	0.047	0.0017
T3 LE → T3 WTC	0.246	0.215	0.072	0.0006
T1 FLA → T3 WTC	−0.241	−0.206	0.062	0.0001
T1 WTC → T3 WTC	0.301	0.319	0.049	<0.001

FLA, Foreign Language Anxiety; IM, Instrumental Motivation; LE, Learning Engagement; WTC, Willingness to Communicate.

### 3.5 Bootstrap mediation effect test

The results of the 5,000-resample bootstrap analysis in Table 6 showed that the independent mediating role of Time 2 instrumental motivation was significant [Estimate = −0.0446, 95% CI (−0.0842, −0.0132)]. The independent mediating role of Time 3 learning engagement was also significant [Estimate = −0.0472, 95% CI (−0.0902, −0.0136)]. Moreover, the sequential mediation involving both Time 2 instrumental motivation and Time 3 learning engagement was significant [Estimate = −0.0163, 95% CI (−0.0329, −0.0049)]. The total indirect effect was −0.1081. This accounted for 31.0% of the total effect. Within this total indirect effect, the instrumental motivation path accounted for 41.3%, the learning engagement path accounted for 43.7%, and the sequential path accounted for 15.1%. Thus, Hypotheses 1, 2, 3, and 4 were supported.

**Table 6:** Bootstrap mediation effect test.

Effect	Est	SE	95% CI	Conclusion
H2: FLA_T1 → IM_T2 → WTC_T3	−0.0446	0.0181	[−0.0842, −0.0132]	Significant
H3: FLA_T1 → LE_T3 → WTC_T3	−0.0472	0.0199	[−0.0902, −0.0136]	Significant
H4: FLA_T1 → IM_T2 → LE_T3 → WTC_T3	−0.0163	0.0073	[−0.0329, −0.0049]	Significant
Total Indirect Effect	−0.1081	0.0275	[−0.1677, −0.0595]	Significant
Total Effect	−0.3492	0.0629	[−0.4705, −0.2256]	Significant
Direct Effect c′	−0.2411	0.0631	[−0.3681, −0.1185]	Significant

### 3.6 Robustness tests

To verify the stability of the model, this study compared alternative mediation models and reverse path models, as presented in Table 7. Alternative Model 1 retained only learning engagement as a mediator. This yielded an indirect effect of −0.0858 [95% CI (−0.1408, −0.0402)]. Alternative Model 2 retained only instrumental motivation as a mediator. This produced an indirect effect of −0.0601 [95% CI (−0.1036, −0.0244)]. The main model presented the largest total indirect effect and included three significant mediation paths. This suggests that the comprehensive sequential model provides more statistical information than the single-mediator models. Furthermore, tests for reverse relationships showed that after controlling for Time 1 foreign language learning anxiety, Time 1 willingness to communicate was not significantly associated with Time 3 foreign language learning anxiety (*B* = −0.023, *β* = −0.028, *p* = 0.605). Similarly, after controlling for Time 2 foreign language learning anxiety, Time 2 instrumental motivation was not significantly associated with Time 3 foreign language learning anxiety (*B* = −0.083, *β* = −0.108, *p* = 0.096). These findings align with the theoretical directions proposed in this study.

**Table 7:** Robustness and diagnostic results.

Test	Result
Alternative Model 1: Only LE as mediator	Indirect effect = −0.0858, 95% CI [−0.1408, −0.0402]
Alternative Model 2: Only IM as mediator	Indirect effect = −0.0601, 95% CI [−0.1036, −0.0244]
Total Indirect Effect of Main Model	−0.1081, 95% CI [−0.1677, −0.0595]
Reverse Path: WTC_T1 → FLA_T3	*B* = −0.023, *β* = −0.028, *p* = 0.605
Reverse Path: IM_T2 → FLA_T3	*B* = −0.083, *β* = −0.108, *p* = 0.096
VIF	1.04–1.28, no severe multicollinearity
Cohen’s *f*^2^	FLA_T1 → IM_T2: 0.071; IM_T2 → LE_T3: 0.131; LE_T3 → WTC_T3: 0.080

## 4 Discussion

This study examined the longitudinal relationships among foreign language learning anxiety, instrumental motivation, learning engagement, and willingness to communicate in vocational English classrooms among Chinese non-English major vocational students. The results showed that foreign language learning anxiety at the beginning of the semester was negatively associated with students’ willingness to communicate at the end of the semester. Instrumental motivation and learning engagement each mediated this relationship, and the sequential pathway from foreign language learning anxiety to instrumental motivation, then to learning engagement, and finally to willingness to communicate was also significant. These findings suggest that vocational students’ willingness to communicate should not be understood only as a direct reflection of language proficiency. Rather, it appears to be embedded in a longitudinal psychological process involving emotion, career-oriented motivation, and classroom engagement (Mohammadzadeh Mohammadabadi, 2025; Zarrinabadi, 2025).

The longitudinal path analysis showed that Time 1 foreign language learning anxiety was significantly and negatively associated with Time 3 willingness to communicate in the vocational English classroom (*β* = −0.206, *p* < 0.001). This supports Hypothesis 1. This finding aligns with the meta-analysis by Teimouri et al. (2019) on the negative correlation between foreign language anxiety and academic achievement. It is also consistent with recent research showing that foreign language anxiety and other negative emotional orientations are closely related to learners’ willingness or unwillingness to communicate in L2 learning contexts (Lin and Wang, 2025; Solhi, 2024). According to the processing efficiency theory (Eysenck et al., 2007), highly anxious individuals involuntarily allocate limited attention and working memory to task-irrelevant internal worries, such as worrying about peers mocking their pronunciation or fearing negative feedback from teachers. In vocational English classrooms, interactive tasks demand a high level of immediate language organization and communication strategies. Therefore, initial anxiety at Time 1 may be associated with cognitive distraction and emotional pressure across the semester, which may help explain why some students report lower readiness to communicate in English at the end of the term. Because classroom silence was not directly measured in this study, the present finding is interpreted in relation to willingness to communicate rather than as direct evidence of classroom silence (Maher and King, 2020).

The study found that Time 2 instrumental motivation for vocational English served as a significant independent mediator between Time 1 anxiety and Time 3 willingness to communicate [Estimate = −0.0446, 95% CI (−0.0842, −0.0132)]. This supports Hypothesis 2. This finding is consistent with the recent perspective of Pekrun’s (2024) control value theory, while also extending the dynamic framework of L2 motivation (Dörnyei and Ushioda, 2011; Papi, 2010). Rather than suggesting that anxiety directly causes motivational decline, the longitudinal evidence indicates that students’ early anxiety was related to their later career-oriented motivational beliefs (Wu, 2024). When vocational students experience negative academic emotions related to fear of making mistakes, fear of negative evaluation, or difficulty understanding classroom input at the beginning of the semester, they may appraise the English classroom as a psychologically threatening environment, thereby heightening the affective filter and reducing their sense of communicative safety (Özdemir and Seçkin, 2025). Over time, this emotional experience may be associated with a weaker perception that vocational English is useful, attainable, or relevant to their future career development. This decline in instrumental motivation associated with anxiety further relates to a decrease in their motivational resources for language communication. It ultimately links to a lower willingness to communicate at the end of the semester (Dong et al., 2022).

Additionally, the independent mediating role of Time 3 learning engagement between Time 1 anxiety and Time 3 willingness to communicate was also significant [Estimate = −0.0472, 95% CI (−0.0902, −0.0136)]. This supports Hypothesis 3. Learning engagement includes behavioral, emotional, and cognitive participation. It acts as a proximal factor connecting motivational beliefs to actual behavior (Fredricks et al., 2004; Zhang and Kim, 2025). High anxiety at Time 1 was significantly and negatively associated with classroom engagement at the end of the semester (*β* = −0.188, *p* < 0.001). Highly anxious individuals often experience physiological arousal and avoidance tendencies during classroom interactions (MacIntyre, 2017). Recent L2 research has also shown that engagement and WTC are closely linked with learners’ achievement emotions, indicating that emotional experiences may be important for understanding different profiles of classroom participation and communication readiness (Wang et al., 2025). Therefore, it is difficult for them to maintain a high level of behavioral focus, positive emotional experiences, and deep cognitive strategies during subsequent classes (Ansari, 2026; Hiver et al., 2021). This continuous disengagement during the learning process relates to a lack of expressive scaffolding and psychological resilience when facing comprehensive vocational tasks at the end of the semester. Consequently, their willingness to communicate remains at a lower level (Wang et al., 2025).

This study outlines the longitudinal chain mediation path where Time 1 foreign language learning anxiety was associated with Time 2 instrumental motivation, which then related to Time 3 learning engagement, and ultimately connected to a lower Time 3 willingness to communicate [Estimate = −0.0163, 95% CI (−0.0329, −0.0049)]. This supports Hypothesis 4. This emotion to motivation to engagement to willingness sequence outlines the dynamic psychological process of lower WTC among vocational students.

This finding aligns with the L2 motivational self system framework (Dörnyei, 2009). For non-English major vocational students, their future professional identities are relatively clear. Their assessment of a course’s usefulness is practical and closely tied to specific vocational tasks. The results show that Time 2 instrumental motivation was positively associated with Time 3 learning engagement (*β* = 0.280, *p* < 0.001). This indicates that instrumental motivation is an important factor linked to higher classroom engagement, echoing recent claims that motivation must manifest as active engagement to influence behavioral outcomes (Henry et al., 2024; Hiver et al., 2021). When initial foreign language anxiety is associated with lower instrumental motivation at Time 2, students may find it more difficult to connect English learning with their imagined future professional identities or ideal L2 selves (Dörnyei, 2009; Papi, 2010). In this state of low instrumental motivation, students’ allocation of behavioral, emotional, and cognitive resources in the classroom declines. This pattern, beginning with emotional experience, passing through career-oriented motivation, and being reflected in classroom engagement, provides a psychological explanation for why vocational students may show limited WTC even when they recognize the practical importance of English (Zare et al., 2025).

## 5 Practical implications

The path coefficients and chain mediation results suggest that supporting vocational students’ willingness to communicate requires attention to emotions, instrumental motivation, and classroom engagement. Relying solely on language skills training is often insufficient.

First, given the direct negative relationship between Time 1 anxiety and subsequent variables (*β* = −0.206), teachers can introduce emotional interventions during the first 4 weeks of the semester. Teachers could move away from high-stakes oral tests at the beginning of the term, choosing instead to provide a low-risk environment with ample instructional support. For instance, teachers can build a customized vocabulary bank for core vocational terms. They can also allow students to complete their initial classroom speaking tasks through low-threat methods such as peer pair work, small group rehearsals, or anonymous voice recordings uploaded to digital learning platforms. Establishing a sense of emotional security early in the course relates to a reduction in the accumulation of anxiety later in the semester.

Second, instrumental motivation plays a meaningful role in the mediation chain. Therefore, traditional teaching materials focused on general grammar and tests often require vocational adaptation to remain relevant to students. Course content can align closely with national occupational standards and international job requirements. For example, mechanical engineering students could practice reading English safety warnings on international manufacturing equipment, while nursing students could practice admission education and pain assessment simulations designed for international wards. Additionally, international tourism students could practice managing complex check-in scenarios and addressing international guest inquiries in English. When anxious students observe that current language tasks are relevant to their future employment, their rational evaluation of the course can help manage negative emotions, which relates to a positive perception of the instrumental motivation of vocational English.

Third, the path analysis shows that Time 3 learning engagement is a direct indicator of willingness to communicate (*β* = 0.215, *p* < 0.001). To translate students’ motivational perceptions into active classroom engagement, vocational English classes can shift away from final evaluations based solely on written exams or random calling. Teachers can implement formative and multi-modal assessments based on industry standards. The evaluation system can incorporate students’ behavioral performance in vocational role-plays, their cognitive reflections on completing simulated workplace tasks, and their emotional support during group collaborations. Reducing penalties for minor grammar mistakes and rewarding the functional completion of vocational tasks can create a supportive environment. This environment encourages anxious students to maintain their engagement, which links to an increased willingness to communicate.

## 6 Limitations and future research

Although this study used a three-wave longitudinal design and controlled for autoregressive paths, there are several academic limitations to address in future research.

First, the study measured learning engagement and willingness to communicate concurrently at Time 3. Although we controlled for prior levels of core variables, reciprocal relationships might exist between engagement and willingness to communicate within the same wave. Future studies can extend the tracking period to four waves or more across an entire academic year. Researchers could utilize a random intercept cross-lagged panel model (RI-CLPM) to analyze the data. This approach helps isolate stable trait differences between individuals and clarifies the temporal sequence from motivational beliefs and engagement to actual communication behavior at the within-person level over time.

Second, all variables were measured using self-report scales. We used Harman’s single-factor test and three-wave confirmatory factor analyses to check for common method bias. However, self-report questionnaires can still be influenced by social desirability bias or recall bias at the end of the semester. Future research can adopt multimodal data fusion approaches to enhance objectivity. Researchers could cross-validate students’ subjective self-reports with objective behavioral metrics. Examples include eye-tracking data during classroom interactions, natural language processing analysis of actual speech output on digital learning platforms, and the duration or pause frequency of pronunciation recorded during peer interactions.

Third, this study treated the participants as a general group of non-English major vocational students. However, actual international participation and the demand for English vary across different vocational fields such as manufacturing, healthcare, and tourism. Future research can include the major category or the clarity of students’ future career identities as a moderator in the model. This extension would test the generalizability and boundary conditions of this chain mediation model across different sub-disciplines, exploring whether strong career goals alter the relationships among anxiety, motivation, and engagement.

Fourth, although this study discussed limited classroom communication in vocational English classrooms, it directly measured willingness to communicate rather than classroom silence. Future studies could include direct measures of classroom silence, reticence, or observed oral participation to examine whether the psychological pathway identified in this study also applies to actual silent behavior or spoken participation in class.

## 7 Conclusion

Based on three-wave longitudinal data, this study highlights the psychological mechanisms through which foreign language learning anxiety relates to willingness to communicate in vocational English classrooms among Chinese non-English major students. The results indicate that foreign language learning anxiety at the beginning of the semester is directly associated with a lower willingness to communicate at the end of the semester. It also shows an indirect relationship by linking to a lower instrumental motivation for vocational English in the middle of the semester and reduced learning engagement at the end of the semester. Instrumental motivation and learning engagement form a significant chain mediation path. The study suggests that lower WTC among vocational students is not merely a single ability issue; rather, it reflects the combined roles of emotions, career-oriented motivation, and classroom engagement. Effective vocational English teaching can aim to manage anxiety, enhance career-related motivational perceptions, and support learning engagement through task-based, supportive, and low-threat classroom designs, which may be associated with improved willingness to communicate in vocational English classrooms.

## Data Availability

The raw data supporting the conclusions of this article will be made available by the authors, without undue reservation.
